# Vascular regression precedes motor neuron loss in the FUS (1-359) ALS mouse model

**DOI:** 10.1242/dmm.040238

**Published:** 2019-08-13

**Authors:** Martin Crivello, Marion C. Hogg, Elisabeth Jirström, Luise Halang, Ina Woods, Megan Rayner, Karen S. Coughlan, Sebastian A. Lewandowski, Jochen H. M. Prehn

**Affiliations:** 1 Department of Physiology and Medical Physics, Centre for the Study of Neurological Disorders, Royal College of Surgeons in Ireland, 123 St. Stephen's Green, Dublin 2, Ireland; 2 FutureNeuro Research Centre, Royal College of Surgeons in Ireland, St. Stephen's Green, Dublin 2, Ireland; 3 Tissue Biology Laboratory, Department of Clinical Neuroscience, Center for Molecular Medicine, Karolinska Institute, Karolinska vägen 8A, 17164, Stockholm, Sweden; 4 Affinity Proteomics, SciLifeLab, School of Biotechnology, KTH-Royal Institute of Technology, Tomteboda vägen 23 A, Stockholm, Sweden

**Keywords:** Amyotrophic lateral sclerosis, FUS, Motoneuron, Angiogenin, Vascular defects

## Abstract

Amyotrophic lateral sclerosis (ALS) presents a poorly understood pathogenesis. Evidence from patients and mutant SOD1 mouse models suggests vascular damage may precede or aggravate motor dysfunction in ALS. We have previously shown angiogenin (ANG) treatment enhances motor neuron survival, delays motor dysfunction and prevents vascular regression in the SOD1^G93A^ ALS model. However, the existence of vascular defects at different stages of disease progression remains to be established in other ALS models. Here, we assessed vascular integrity *in vivo* throughout different disease stages, and investigated whether ANG treatment reverses vascular regression and prolongs motor neuron survival in the FUS (1-359) mouse model of ALS. Lumbar spinal cord tissue was collected from FUS (1-359) and non-transgenic control mice at postnatal day (P)50, P90 and P120. We found a significant decrease in vascular network density in lumbar spinal cords from FUS (1-359) mice by day 90, at which point motor neuron numbers were unaffected. ANG treatment did not affect survival or counter vascular regression. Endogenous *Ang1* and *Vegf* expression were unchanged at P50 and P90; however, we found a significant decrease in miRNA 126 at P50, indicating vascular integrity in FUS mice may be compromised via an alternative pathway. Our study demonstrates that vascular regression occurs before motor neuron degeneration in FUS (1-359) mice, and highlights that heterogeneity in responses to novel ALS therapeutics can already be detected in preclinical mouse models of ALS.

This article has an associated First Person interview with the joint first authors of the paper.

## Abstract (precis)

**Summary:** Vascular regression is observed prior to motor neuron loss in the FUS (1-359) mouse model of ALS, yet is not rescued by angiogenin treatment.

## INTRODUCTION

Amyotrophic lateral sclerosis (ALS) is a neurodegenerative disease characterised by progressive motor neuron death, leading to muscle weakening and motor-function loss. Time to death after diagnosis averages 3-5 years, usually as a result of respiratory failure (Robberecht and Philips, 2013). Its aetiology remains largely enigmatic. Out of dozens of clinical trials, only two drugs have become medically approved, riluzole and recently the antioxidant edavarone (Petrov et al., 2017). Both are of limited effect, extending lifespan by several months and enhancing motor-function outcomes in a subset of patients, respectively (Rothstein, 2017). This probably stems from the heterogeneous nature of the disease, in which patients can display wide differences in onset time and location, rate of decline and survival time (Chio et al., 2009; Pupillo et al., 2014; Turner et al., 2013).

There is a growing body of evidence that suggests ALS could be treated as a neurovascular disease (Garbuzova-Davis et al., 2011). Indeed, a recent review highlights that neurovascular changes occur in many neurodegenerative diseases, indicating that this pathway could be functionally relevant across the spectrum of disorders (Sweeney et al., 2018). SOD1^G93A^ mutant mice have been shown to have compromised endothelia prior to disease onset (Zhong et al., 2008; Winkler et al., 2014), and reduced vascular perfusion has been reported in the central nervous system of ALS mice and patients (Zhong et al., 2008; Rule et al., 2010; Winkler et al., 2013). Loss-of-function mutations in angiogenin (ANG) have been shown to segregate with ALS patients (van Es et al., 2009; Greenway et al., 2006; Wu et al., 2007). Angiogenin is a potent angiogenic factor that is also capable of initiating stress responses (Yamasaki et al., 2009). Reduced *Ang1* and vascular endothelial growth factor family (*Vegf*) gene expression has been reported in SOD1^G93A^ mice (Lu et al., 2007). All this has stimulated research exploring the therapeutic potential of VEGF by others (Keifer et al., 2014; Pronto-Laborinho et al., 2014) and of ANG by our group (Kieran et al., 2008; Sebastia et al., 2009). Most recently, our lab conducted a SOD1^G93A^ mouse model study following the guidelines set by Ludolph et al. for ALS preclinical studies (Ludolph et al., 2010). Among the more salient results, we found that systemic human (hu)ANG administration three times a week from symptom onset extended survival, delayed motor dysfunction and protected against motor neuron loss and vascular regression (Crivello et al., 2018).

These preclinical guidelines further recommend the advancement of ALS models other than the SOD1^G93A^ ‘gold standard’ (Ludolph et al., 2010). One such model is FUS transgenic mice. Here, we employed FUS (1-359) transgenic mice, which carry a truncated version of FUS with no nuclear localisation signal (NLS) (Shelkovnikova et al., 2013). These mice present severe motor degeneration starting ∼P107 (range 60-180) and display a rapid rate of progression, reaching terminal disease stage within several days after symptom onset (Shelkovnikova et al., 2013; Hogg et al., 2018). We have characterised the evolution of vascular defects in relation to motor neuron degeneration at different disease time points and tested the potential of ANG treatment in the FUS (1-359) model.

## RESULTS

### Motor neuron loss in the FUS (1-359) mouse model

**Fig. 1.**
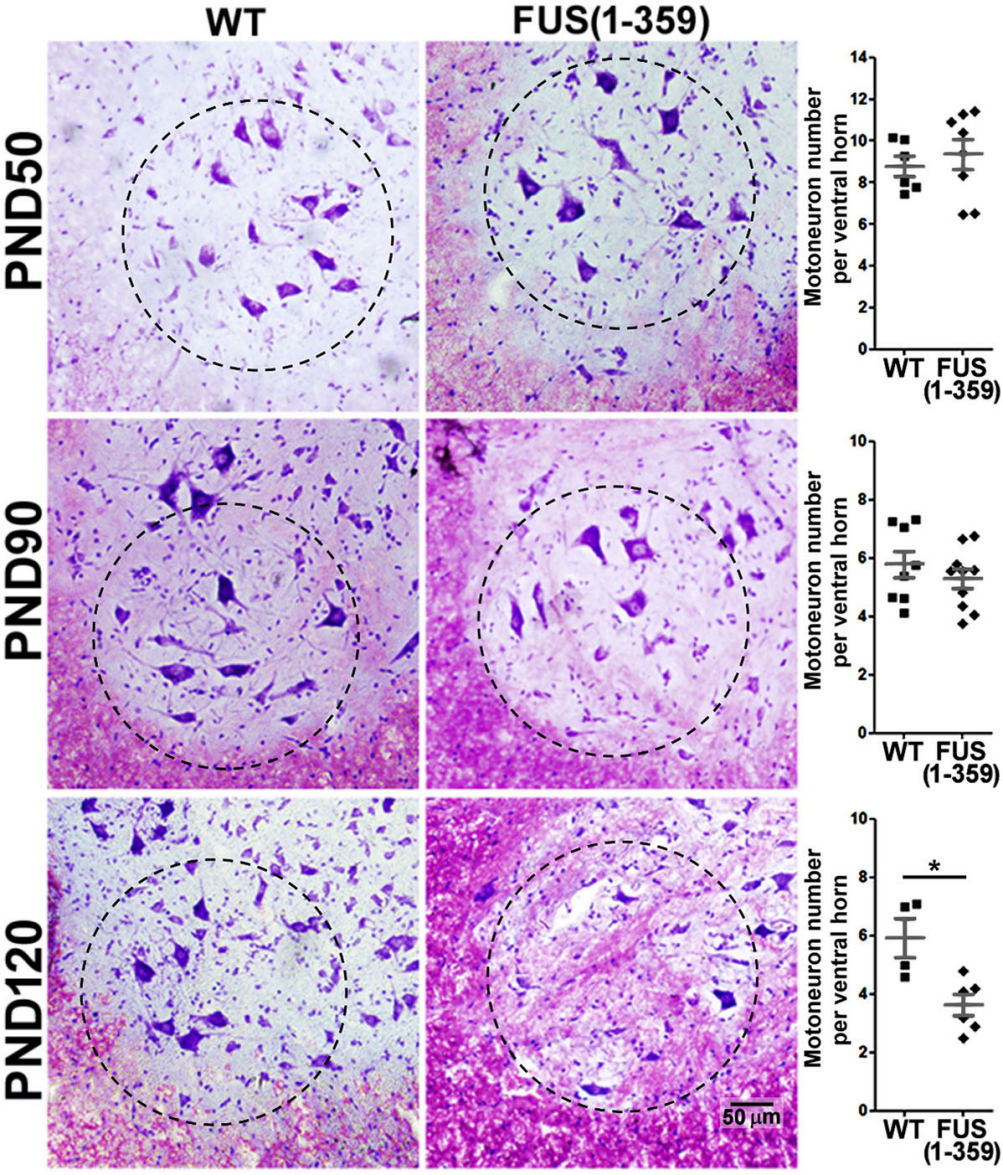
**Nissl staining revealed significant motor neuron loss by P120 in FUS (1-359) mice.** Nissl stain highlights nucleic acid, in particular ribosomal RNA, which is abundant in motor neurons and results in a dark purple stain of the cell body. Figure shows representative images from the ventral horn area of spinal cord tissue from WT and FUS (1-359) transgenic mice, accompanied by the quantification scatter-plot graph, obtained at P50 [top; WT *n*=6, FUS (1-359) *n*=8], P90 [middle; WT *n*=8, FUS (1-359) *n*=10] and P120 [bottom; WT *n*=4, FUS (1-359) *n*=6]. Dashed circle indicates ventral horn region used for quantification. Each datapoint represents mean motor neuron counts from 20 non-consecutive tissue slices along the lumbar spinal cord, dark grey lines represent mean±s.e.m. *P=0.005 [*t*-test: WT versus FUS (1-359) at P120].

We first profiled motor neuron numbers in the lumbar spinal cord from a pre-symptomatic time point (P50), around onset (P90), to the mid-to-late stages of disease (P120). As expected, P50 FUS (1-359) mice showed no motor neuron loss (Fig. 1). We also found no significant differences in motor neuron counts between transgenic mice and their wild-type (WT) counterparts at P90. In contrast, by P120 we found a significant decrease in motor neuron numbers in FUS (1-359) mice compared to WT littermates in the ventral horn of the lumbar spinal cord [Fig. 1; FUS (1-359): 3.61±0.36 (mean±s.e.m.) versus WT: 5.95±0.66; *P*<0.005].

### Vascular regression precedes motor neuron loss in FUS (1-359) mice

**Fig. 2.**
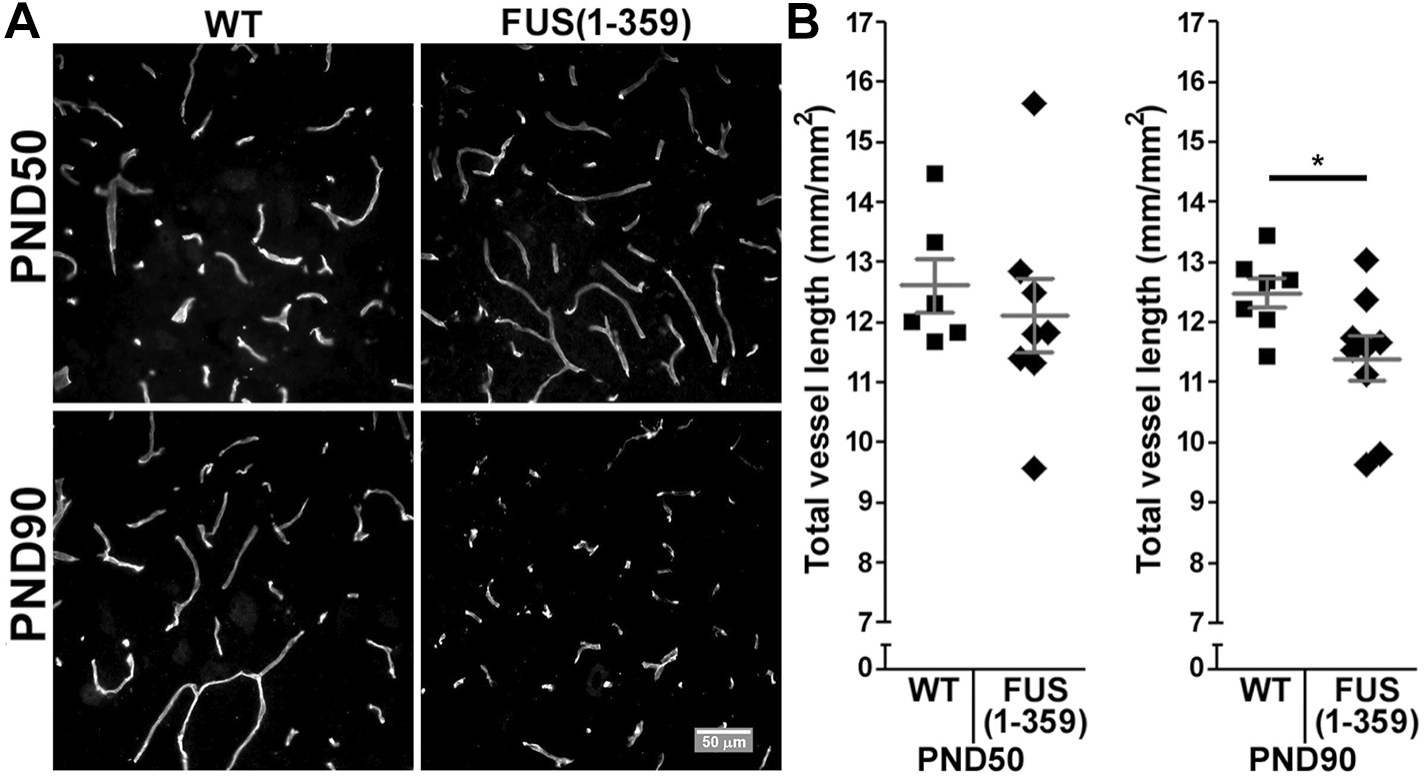
**Analysis of podocalyxin immunostaining revealed vascular regression by P90 in FUS (1-359) mice.** (A) Representative images from the ventral horn area of lumbar spinal cord tissue from WT (left) and FUS (1-359) (right) transgenic mice obtained at P50 (top) and at P90 (bottom). (B) Scatter-plot graphs showing the quantified total vessel length per lumbar ventral horn area at P50 [left; WT *n*=6, FUS (1-359) *n*=8] and P90 [right; WT *n*=7, FUS (1-359) *n*=9]. Each datapoint represents mean vessel length from six non-consecutive tissue slices along the lumbar spinal cord, dark grey lines represent mean±s.e.m. *P<0.04 [*t*-test: WT versus FUS (1-359) at P90].

We next analysed total vessel length per area of ventral horn grey matter as a metric of vascular integrity by immunostaining against podocalyxin, an endothelial cell marker in lumbar spinal cord sections. We observed no differences in the average total length of the lumbar ventral horn capillary network between WT and FUS (1-359) mice at P50 (Fig. 2). Interestingly, we found a significant decrease in average vessel length per ventral horn in the transgenic mice by P90 [Fig. 2; FUS (1-359): 11.40±0.37 mm/mm^2^ versus WT: 12.49±0.25 mm/mm^2^; *P*<0.04].

**Fig. 3.**
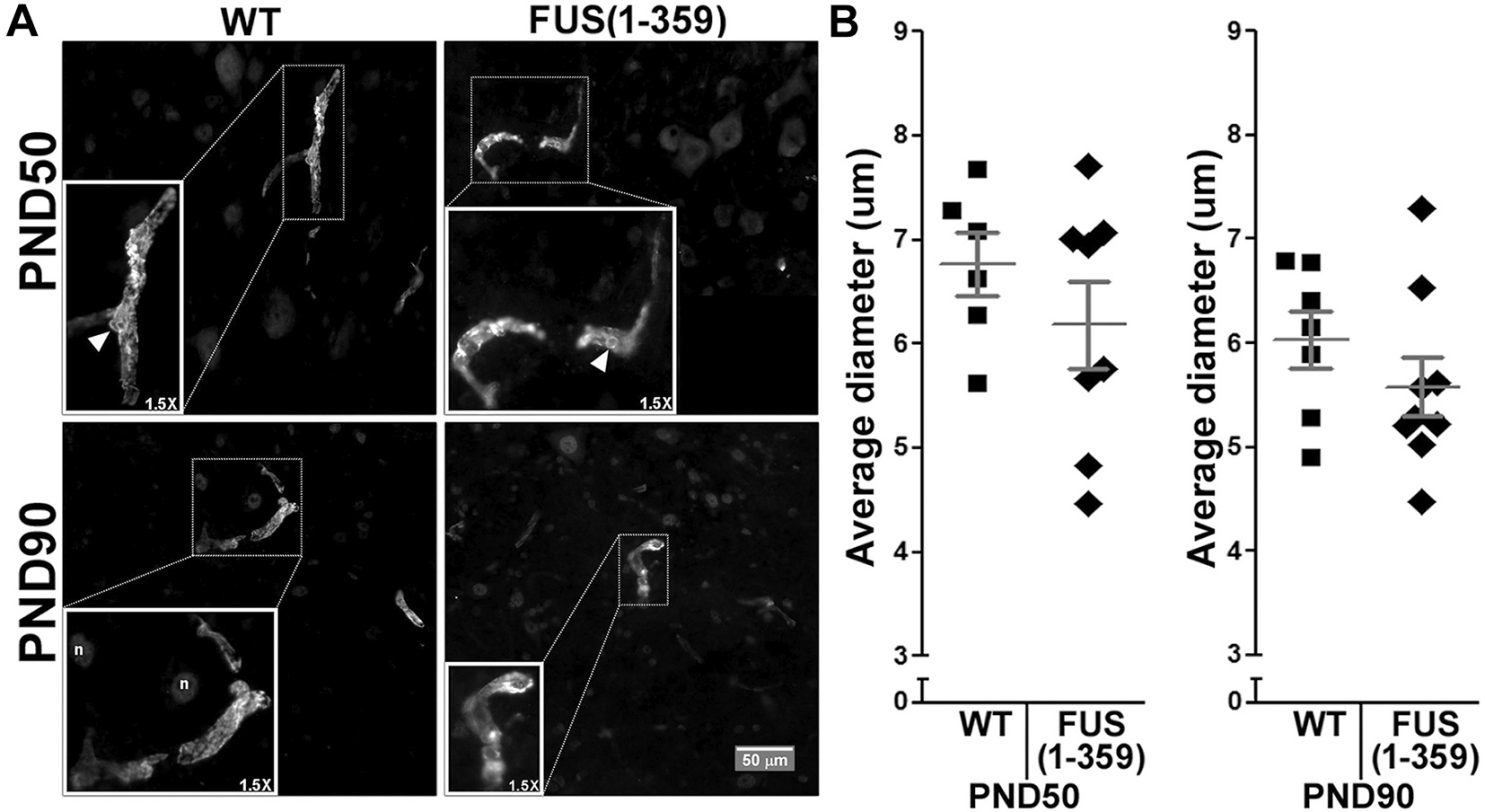
**No differences in diameter identified when analysing ASMA^+^ blood vessels.** (A) Representative images from the ventral horn area of lumbar spinal cord tissue from WT (left) and FUS (1-359) (right) transgenic mice obtained at P50 (top) and at P90 (bottom). Insets show magnified view of boxed areas; arrows indicate pericyte cell bodies; n indicates nuclei. (B) Scatter-plot graphs showing the quantified diameter of ASMA^+^ blood vessels per lumbar ventral horn at P50 [left; WT *n*=6, FUS (1-359) *n*=8] and P90 [right; WT *n*=7, FUS (1-359) *n*=9]. Each datapoint represents mean diameter from six non-consecutive tissue slices along the lumbar spinal cord, dark grey lines represent mean±s.e.m. *t*-test between FUS (1-359) and WT groups non-significant in all cases.

We have previously identified a significant increase in the average diameter of alpha-smooth muscle actin positive (ASMA^+^) blood vessels in the SOD1^G93A^ mouse model (Crivello et al., 2018). Therefore we also immunostained the tissues with an antibody to ASMA, a marker for the blood vessel-surrounding contractile cells (i.e. smooth muscle cells and pericytes). We speculated that increased diameter of vessels could be a compensatory mechanism to counter vascular regression. However, we found no differences between WT and FUS (1-359) mice at either P50 or 90 when analysing ASMA^+^ vascular diameter (Fig. 3), highlighting significant heterogeneity in vascular pathology among preclinical ALS disease models.

### ANG treatment did not extend survival or delay symptom onset in FUS (1-359) mice

**Fig. 4.**
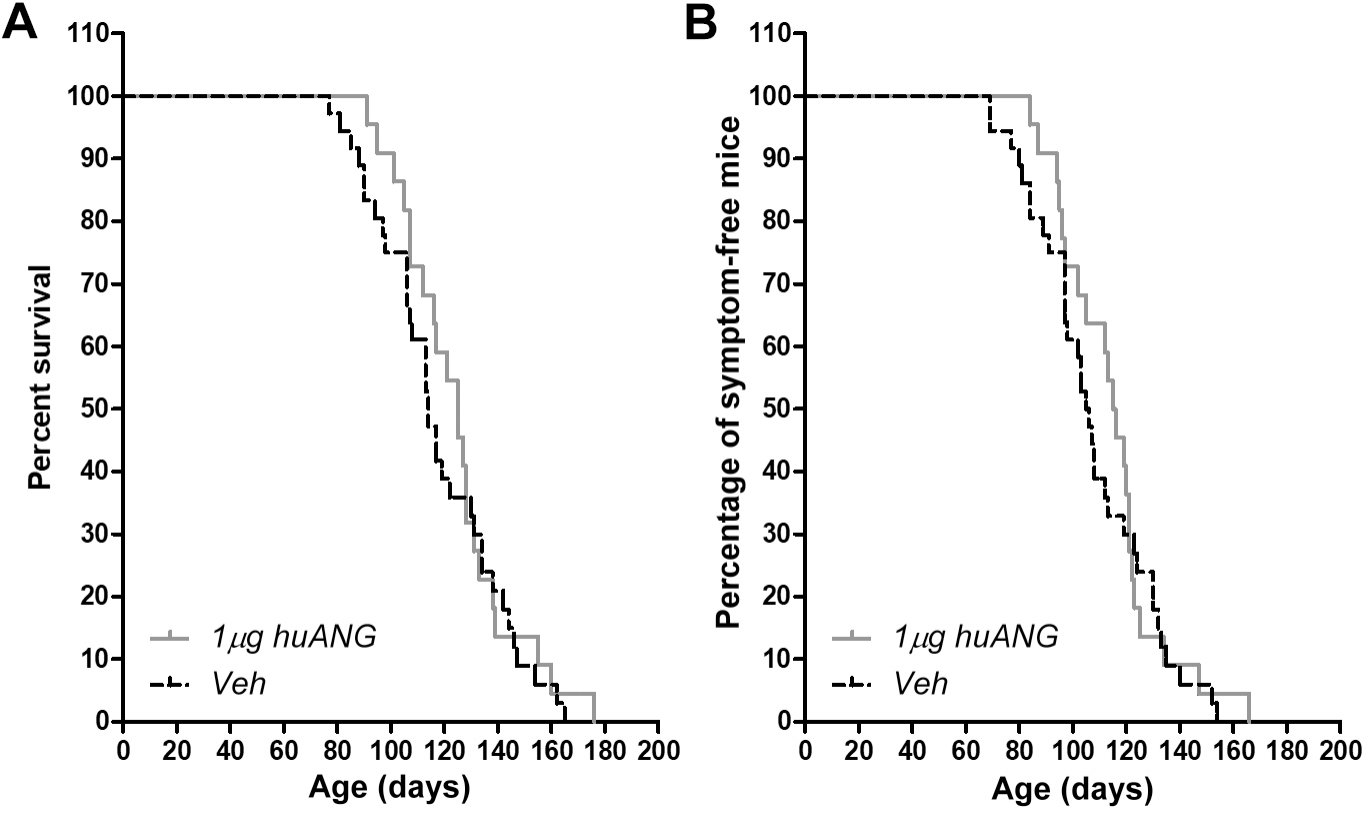
**No differences in survival or time of disease onset in FUS (1-359) mice treated with huANG.** (A) Kaplan–Meier curves showing survival rates from animals treated three times per week from P50 with either 1 μg of huANG (grey line) or PBS vehicle (dashed black line). (B) Kaplan–Meier curves showing time of disease onset measured in animals treated three times per week from P50 with either 1 μg of huANG (grey line) or PBS vehicle (dashed black line). Log-rank test showed no significant differences between treated and control groups in both cases (*n*=22 huANG, *n*=25 PBS vehicle).

Following the vascular results described above and our previous finding in the SOD1 mouse model (Crivello et al., 2018), we next explored the effects of chronic huANG administration on survival and disease onset. We adapted the pre-clinical study design previously reported by us, and treated *n*=22-25 FUS (1-359) mice chronically three times a week with 1 μg huANG or vehicle until each mouse reached terminal disease stage and was terminally anesthetised. Interestingly, log-rank analysis revealed no significant differences between sex- and litter-matched FUS (1-359) mice treated chronically three times a week with 1 μg huANG versus vehicle in terms of survival (Fig. 4A) or time of symptom onset (Fig. 4B).

### ANG treatment did not prevent vascular regression or affect motor neuron numbers

**Fig. 5.**
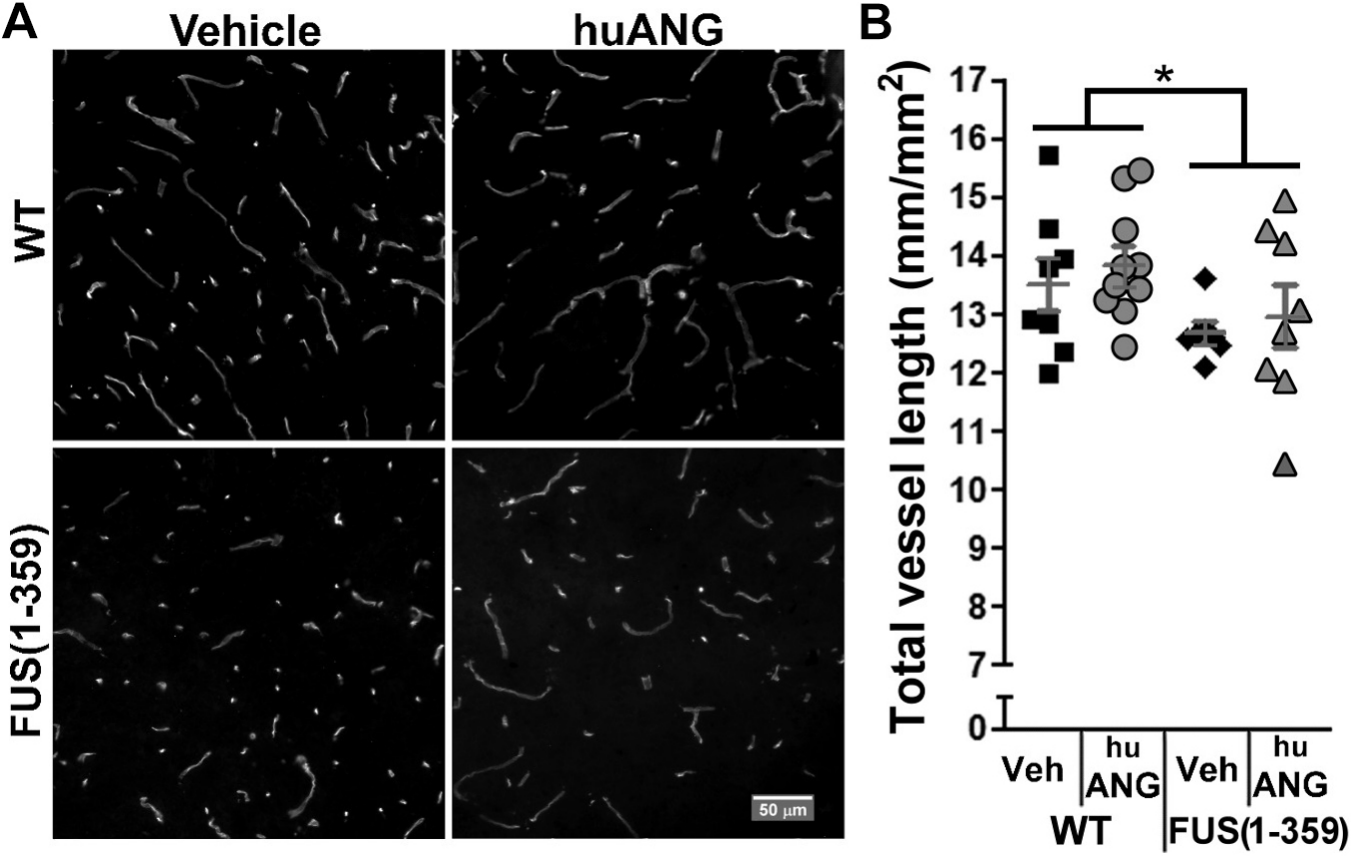
**huANG treatment did not counteract vascular regression.** (A) Representative images from the ventral horn area of lumbar spinal cord tissue from vehicle- (left) and huANG- (right) treated WT mice (top) and FUS (1-359) mice (bottom) obtained at P70 following 20 days treatment and stained with an antibody to podocalyxin to highlight blood vessels. (B) Scatter-plot graph showing the quantified total vessel length per lumbar ventral horn area at P70 obtained from the four groups of mice. Each datapoint represents mean vessel length from six non-consecutive tissue slices along the lumbar spinal cord. WT-huANG *n*=10; WT-Veh *n*=8; FUS (1-359)-huANG *n*=8; FUS (1-359)-Veh *n*=7. Dark grey lines represent mean±s.e.m. *P<0.04, main effect genotype; interaction and main effect treatment, not significant (two-way ANOVA).

We also conducted a vascular analysis as described above in lumbar sections from sex- and litter-matched WT and FUS (1-359) mice treated chronically three times a week with 1 μg ANG from P50 to P70. We found FUS (1-359) mice to have a significantly reduced capillary network compared to their WT counterparts, which was unaffected by treatment [Fig. 5; two-way ANOVA; interaction: not significant (ns); main effect treatment: ns; main effect genotype: *P*<0.04].

**Fig. 6.**
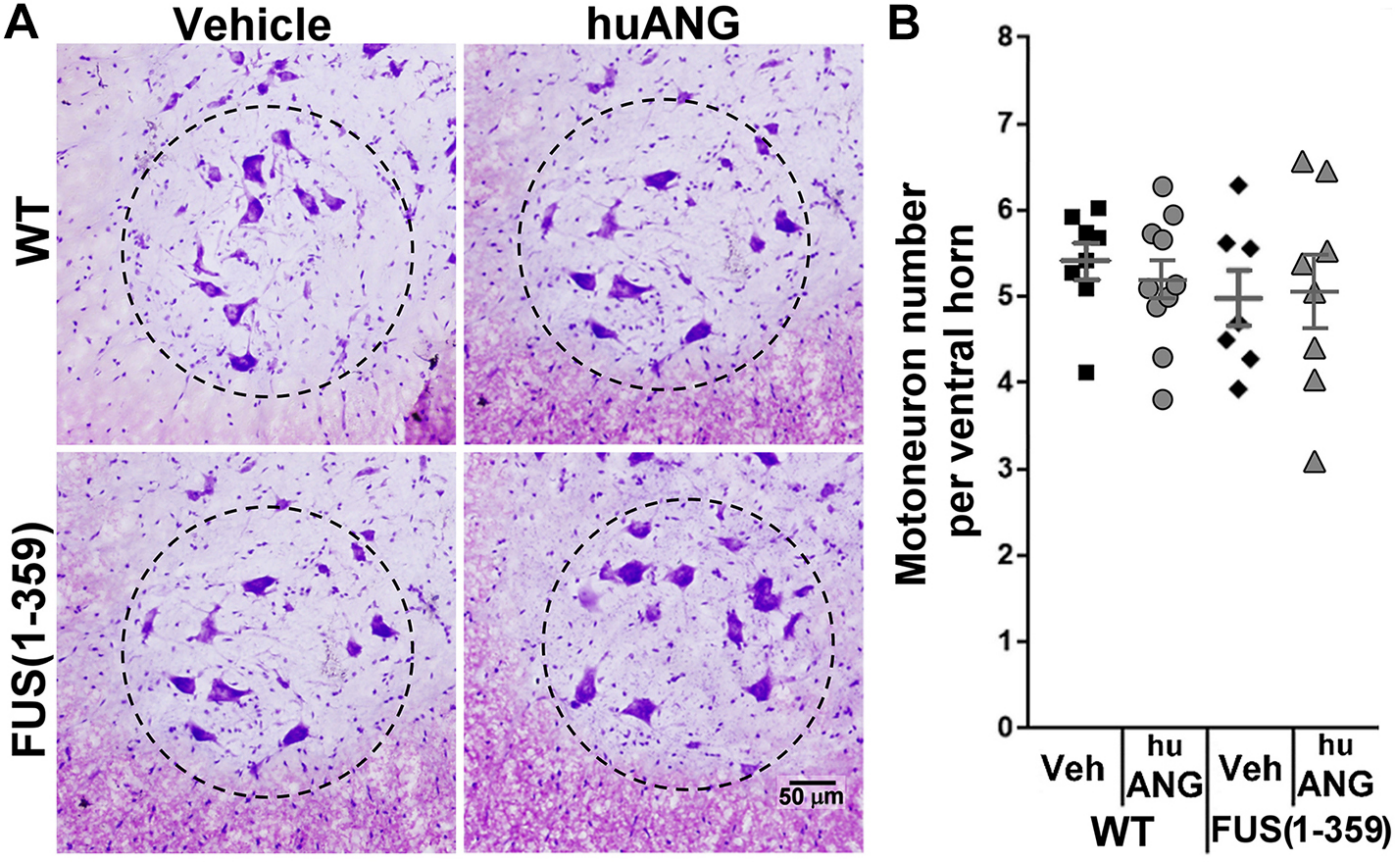
**huANG treatment did not affect expected motor neuron numbers.** (A) Representative images of Nissl-stained motor neurons in the ventral horn area of lumbar spinal cord tissue obtained at P70 from vehicle (left)- and huANG (right)-treated WT (top) and FUS (1-359) (bottom) mice. Dashed circle indicates ventral horn region used for quantification. (B) Scatter-plot graph showing the quantified number of motor neurons per lumbar ventral horn area at P70 obtained from the four groups of mice. Each datapoint represents mean number of motor neurons from 20 non-consecutive tissue slices along the lumbar spinal cord. WT-huANG *n*=10; WT-Veh *n*=8; FUS (1-359)-huANG *n*=8; FUS (1-359)-Veh *n*=7. Dark grey lines represent mean±s.e.m. Interaction, main effect treatment and main effect genotype, not significant (two-way ANOVA).

We also Nissl stained tissues from this same group of spinal cord samples treated with huANG until P70 (Fig. 6). We found no significant differences in the number of motor neurons at this time point between the two genotypes or between treatments (Fig. 6; two-way ANOVA; interaction: ns; main effect treatment: ns; main effect genotype: ns).

### m*Ang1* expression is not altered in FUS (1-359) mice

**Fig. 7.**
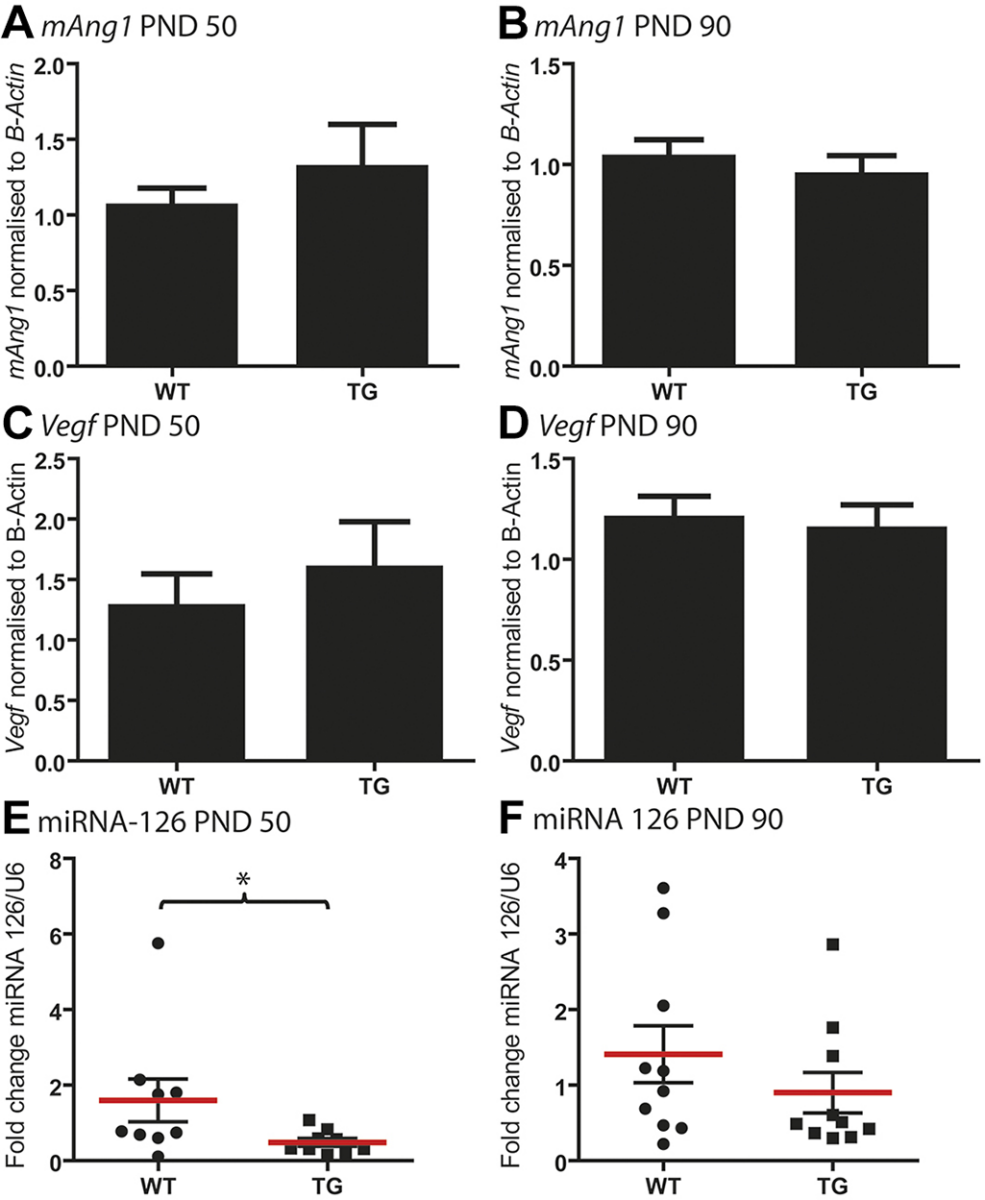
**miRNA 126 is significantly downregulated in FUS (1-359) mice at P50.** (A,B) qPCR on *Ang1* levels in spinal cord RNA from mice at P50 (A) and P90 (B) revealed no significant change in *Ang1* levels (*n*=7-9 mice/group). (C,D) qPCR to quantify *Vegf* mRNA in spinal cord also showed no significant difference in levels at P50 (C) and P90 (D). (E,F) Taqman assays were used to quantify miRNA 126 levels in total RNA extracted from spinal cord at P50 (E) and P90 (F) (*n*=7-9 mice/group). At P50 miRNA 126 levels were significantly lower in transgenic (TG) FUS (1-359) mice compared to WT. *P<0.05 (Mann–Whitney U test), whereas no significant difference was detected at P90. Data are mean±s.e.m.

Quantification of mouse (m)*Ang1* (*Ang*) levels by qPCR on lumbar spinal cord RNA revealed no significant difference in endogenous m*Ang1* levels when comparing WT to FUS (1-359) littermates at P50 or P90 (*n*=9-10 mice/group; Fig. 7A,B). Similarly, analysis of *Vegf* (*Vegfa*) mRNA levels by qPCR revealed that there was also no significant difference when comparing WT to FUS (1-359) littermates at P50 or P90 (Fig. 7C,D). To further explore potential mechanisms for the lack of beneficial effect of angiogenin we investigated levels of miRNA 126 in early disease stages, as it has previously been implicated in vascular disease via regulation of angiogenesis (Jamaluddin et al., 2011). miRNA 126 is the most highly abundant miRNA in endothelial cells and knockdown of miRNA 126 in endothelial cells rendered them unresponsive to VEGF-mediated proliferation and wound repair (Fish et al., 2008). Here, we found that levels of miRNA 126 were significantly decreased in spinal cord from FUS (1-359) mice compared to WT mice at P50 (Fig. 7E), but no significant difference was detected at P90 (Fig. 7F). This early downregulation of miRNA 126 may render FUS (1-359) mice unable to respond to pro-angiogenic signalling induced by angiogenin in the early stages of disease progression.

In conclusion, we find the FUS (1-359) transgenic mice show depletion of miRNA 126 at a very early stage of disease (P50), which appears to limit their ability to respond to endogenous or exogenous angiogenic factors.

## DISCUSSION

Several genes have been linked directly or indirectly to ALS in recent years (Chia et al., 2018; Picher-Martel et al., 2016), allowing for the generation of multiple new transgenic models and the need to properly characterise them. Of particular interest here, mutations in the highly conserved NLS region of the *FUS* gene, which encodes a protein that is involved in RNA transcription and processing, have been described in ALS and in ALS with frontotemporal dementia (ALS/FTD) (Dormann and Haass, 2013; Hewitt et al., 2010; Nolan et al., 2016; Rademakers et al. 2010). FUS (1-359) mice express a version of the FUS protein that lacks its entire NLS and RNA-binding motifs, but they share several phenotypic hallmarks with ALS-FUS patients, who predominantly present an aggressive clinical course, severe neuronal loss in the spinal cord ventral horn (Ravits et al., 2013), and mislocalisation and aggregation of FUS protein in the cytoplasm (Shelkovnikova et al., 2013; Hewitt et al., 2010).

In the present study, we characterised vascular integrity in the FUS (1-359) mouse model at different time points in the disease course. We found a significant reduction in vascular coverage per ventral horn grey matter area in the lumbar spinal cord at P70 (Fig. 5), and P90 (Fig. 2), but only by the next time point analysed (P120) did we observe a significant loss of motor neurons (Fig. 1). This shows that vascular regression precedes motor neuron loss in this model, as has been previously described in mutant SOD1 mice as assessed by immunoglobulin leakage and haemosiderin deposition within the spinal cord (Zhong et al., 2008). However, our subsequent studies pointed at potential differences in vascular regression in SOD1 and FUS mutant mice. We previously demonstrated that early vasculature defects in SOD1^G93A^ mice could be rescued by huANG administration from P90, which prevented later motor neuron loss (analysed at P115, following 25 days of treatment) (Crivello et al., 2018). Using a similar treatment protocol, huANG administration exerted no beneficial effects on vascular regression, motor neuron loss or survival in FUS (1-359) mice. We also failed to detect an increase in the average diameter of vascular mural cells in FUS (1-359) mice. We detected such an increase in transgenic SOD1^G93A^ mice at an early-to-mid point in the disease (Crivello et al., 2018). We proposed this to be a possible compensatory mechanism in response to reduced vascular network coverage. Collectively, the SOD1^G93A^ and FUS (1-359) ALS mouse models show fundamental differences in terms of vascular pathophysiology, which may correlate to ANG responsiveness. Of note, a previous study in the SOD1^G93A^ ALS mouse model demonstrated that treatment of mice with an activated protein C (APC) mutant (5A-APC) protected blood-spinal cord barrier integrity and delayed onset of motor symptoms (Winkler et al., 2014), indicating there are multiple effective methods for targeting vascular defects in ALS.

To explain these differences in vascular pathology and responses to pro-angiogenic therapies in both mouse models, we highlight one aspect of FUS mutant protein: loss of nuclear function in endothelia. FUS has been shown to be expressed in human endothelia from multiple organs (Andersson et al., 2008). Furthermore, studies using different mammalian endothelial cells in culture have described how FUS levels are downregulated as the cells transition from proliferation to quiescence (Alliegro and Alliegro, 1996), and the use of antibodies or siRNA against FUS protein or mRNA inhibited their proliferation (Alliegro and Alliegro, 2002; Yoshida et al., 2010). Given that FUS affects the transcription and/or processing of multiple RNAs in multiple cell types (Fujioka et al., 2013; Lagier-Tourenne et al., 2012) we should not ignore FUSopathic effects in neurons, glia or muscle; however, the evidence described suggests that dysfunctional FUS can send vascular homeostasis into disarray, even when ignoring the toxic potential of FUS cytoplasmic inclusions. We hypothesise that FUS (1-359) mice may be lacking an important functionality directly related to endothelial cell proliferation, which would explain why huANG treatment did not have the positive effects we have seen before in SOD1^G93A^ mice. To our knowledge, this is the first study into vasculature defects in the FUS (1-359) mouse model. Several other FUS ALS mouse models have been developed; however, vasculature analysis and blood-brain barrier (BBB) defects have not been explored in these models to date (Devoy et al., 2017; Scekic-Zahirovic et al., 2017). BBB defects are implicated in multiple neurodegenerative diseases, which indicates that defects in this pathway may yield therapeutic targets of wide relevance (Sweeney et al., 2019).

The SOD1^G93A^ mouse model displays downregulated *Vegf* mRNA early in disease progression (Lu et al., 2007), and suffers a more gradual disease progression than the FUS (1-359) mice (Hogg et al., 2018). To further explore the role of FUS in the vasculature system we analysed levels of pro-angiogenic factors *Vegf* and *Ang1* by qPCR. We found no significant difference in expression of these factors between WT and FUS (1-359) mice at early time points when we observed vasculature defects. We also investigated miRNA 126, as it has previously been implicated in vascular disease via regulation of angiogenesis (Jamaluddin et al., 2011). In endothelial cells, miRNA 126 has been shown to promote proliferation and enhance vascular repair following injury to the carotid artery (Schober et al., 2014). miRNA 126 has been shown to negatively regulate levels of SPRED1 and PIK3R2, which prevents downstream signalling in response to VEGF via the MAP kinase and the PI3 kinase pathways, respectively (Fish et al., 2008; Wang et al., 2008). Interestingly, we found that miRNA 126 was significantly downregulated in FUS (1-359) mice early in disease, indicating a profound early defect in angiogenesis signalling in the spinal cord. This defect could be related to the more aggressive pathology observed in FUS (1-359) mice. Interestingly, miRNA 126 has been shown to be downregulated early in disease progression in muscle from the SOD1^G93A^ mouse model (Maimon et al., 2018), and the authors showed that overexpressing miRNA 126 could prevent axonal degeneration and loss of innervation at the neuromuscular junction. This raises the possibility that increasing miRNA 126 levels may delay onset of symptoms in the FUS (1-359) mouse model. FUS has been associated with numerous stages of RNA processing involving both coding and non-coding RNAs (reviewed by Lagier-Tourenne et al., 2010). FUS interacts with argonaute 2 and directly binds to miRNAs (Zhang et al., 2018); however the FUS (1-359) fragment expressed in this mouse colony lacks the RNA-binding region required to interact directly with RNA, which indicates that the observed decrease in miRNA 126 occurs via an indirect mechanism. In conclusion, our results highlight the importance of incorporating multiple models in preclinical ALS studies to more accurately approximate the full range of ALS clinical manifestations and effects of novel therapeutics (such as huANG), and thus provide a better basis for clinical trials.

## MATERIALS AND METHODS

### Animals and ethics approval

This study was conducted in strict accordance with Directive 2010/63/EU on the protection of animals used for scientific purposes. FUS (1-359) mice were generated in the laboratory of Prof. Vladimir Buchman (Cardiff University; Shelkovnikova et al., 2013) and re-derived at the Institute of Molecular Genetics of the ASCR, Prague, Czechia. Mice for this study were age-, gender- and litter-matched according to ALS pre-clinical trial guidelines (Ludolph et al., 2010). The animals, congenic on the C57Bl/6 background, were housed in cages of between 3 and 5 mice at a constant temperature (22°C) on a 12 h light/dark cycle (07:00 h on, 19:00 h off), with *ad libitum* availability of food and water. Exact numbers of individuals used are detailed in Table S1 and S2. Mice were genotyped by PCR. Ethical approval for the animal experiments conducted for this study was awarded by the Animal Research Ethics Committee of the Royal College of Surgeons in Ireland (ethics reference number: REC1122b) and under license from the Irish Health Products Regulatory Authority (AE19127/P004).

### Tissue collection and preparation for histological analysis

Following ethical guidelines, mice at P50, P90 and P120 were terminally anesthetised and transcardially perfused via the left ventricle with PBS at a slow rate (∼10 ml/min) until full exsanguination. Lumbar spinal cords (L1-L5) were collected and fixed with PFA 4% for 1 h before being transferred to a 30% sucrose solution for cryoprotection. After 1-5 days, the sucrose solution was completely removed, the tissues frozen in liquid nitrogen and stored at −80°C. The tissues were later sectioned with a cryostat set at 16 μm and mounted on Superfrost plus slides (MNJ-700-010N, VWR).

### Vascular immunostaining and stereological analysis

We conducted immunohistochemistry against two markers that we had analysed previously to assess vascular integrity: podocalyxin (endothelial cell marker) and ASMA (expressed in contractile mural cells) (Crivello et al., 2018). The tissue sections were washed in PBS and then permeabilised using 3% triton-PBS for 20 min. Blocking was conducted with 5% donkey serum for 1 h before incubating the tissues overnight with 1:200 anti-podocalyxin antibody (AF1556, R&D Systems) and 1:50 anti-ASMA (ab5694, Abcam). The next day the samples were washed and incubated with anti-goat Alexa-647 (A21447, Thermo Fisher Scientific) and anti-rabbit Alexa-488 (A10042, Thermo Fisher Scientific) secondary antibodies 1:200 for 1 h at room temperature. Finally, the slides were mounted with DAPI-containing media (H1200, Vector Labs).

Pictures fitting grey matter from the left and right ventral horn were captured using a Nikon TE2000s epifluorescence microscope at 20× magnification. The images were analysed using ImageJ software by a blinded observer as described previously (Lewandowski et al., 2016). Briefly, a common threshold selection was used to generate masks and the ‘skeletonisation’ function was then used for measuring blood vessel length. ASMA^+^ blood vessel diameter was calculated by dividing the total mask area of each continuous ASMA signal by their total length. Each data point graphed represents the average from at least six non-adjacent tissue sections (>120 μm apart).

### RNA extraction and qPCR

Mice were transcardially perfused as described above and tissues dissected then flash frozen in liquid nitrogen. Total RNA was extracted using the Qiagen miRNeasy kit and protocol. For gene expression qPCR, 500 ng total RNA was reverse transcribed with oligo dT primers and amplified with *Ang1* or *Vegf* primers (from Sebastia et al., 2009) using Quantitect SYBR green PCR kit (Qiagen). For miRNA analysis, total RNA was analysed using the Taqman assay mmu-126-5p and normalised to U6 snRNA (Applied Biosystems). Fold change was calculated using the 2*^−DDCt^* method (Livak and Schmittgen, 2001).

### Motor neuron counts with Nissl staining

Motor neuron survival was assessed from the same group of tissues described above. First, the samples were treated with 0.1% Cresyl Violet acetate solution for 20 min at 60°C before being rinsed with distilled water and treated with increasing concentrations of ethanol (70%, 90% and 100%). A 1 min treatment with HistoClear (HS200, National Diagnostics) was the final step, before mounting the slides with DPX mounting medium. Nissl-stained motor neurons of the ventral horn region were counted following previously published methods (cell bodies ≥30 μm in size, presence of a dark nucleolus and multi-polar structure) (Coughlan et al., 2015) in 20 non-consecutive sections per animal.

### ANG treatment

Following results that we had obtained previously in SOD1^G93A^ mice, we next pursued a similar recombinant huANG (265AN250/CF, R&D Systems) treatment paradigm with FUS (1-359) mice (Crivello et al., 2018). FUS (1-359) mice and their WT counterparts were injected intraperitoneally with 1 µg of huANG or vehicle (PBS) three times a week from P50 until P70 for histological studies or from P50 until end stage of disease for survival analysis. Disease end stage was determined by the extreme weakening or loss of hind limb function and inability of the mice to turn themselves within 30 s of being placed on their back (Ludolph et al., 2010).

### Statistical analysis

Outliers classed as ±2 s.d. from the mean were excluded from further analysis. Two-tailed *t*-tests were used to analyse differences in the means between groups of two. Two-way ANOVA was used for groups of four testing for factors of genotype and of treatment. Mantel-Cox test was used to analyse differences in lifespan measurements. Normality was assessed with the Shapiro-Wilk test and homoscedasticity with the Levene test.

## Supplementary Material

Supplementary information
