# Correction: Vascular regression precedes motor neuron loss in the FUS (1-359) ALS mouse model

**DOI:** 10.1242/dmm.045310

**Published:** 2020-05-15

**Authors:** Martin Crivello, Marion C. Hogg, Elisabeth Jirström, Luise Halang, Ina Woods, Megan Rayner, Karen S. Coughlan, Sebastian A. Lewandowski, Jochen H. M. Prehn

There was an error in Disease Models & Mechanisms (2019) 12, dmm040238 (doi:10.1242/dmm.040238).

The authors mistakenly used the wrong image for the FUS(1-359) vehicle panel in Fig. 6A when assembling the figure. The corrected and original figure panels are shown below. The quantification of data shown in Fig. 6B is not affected by this error.

**Fig. 6A (original panel).**
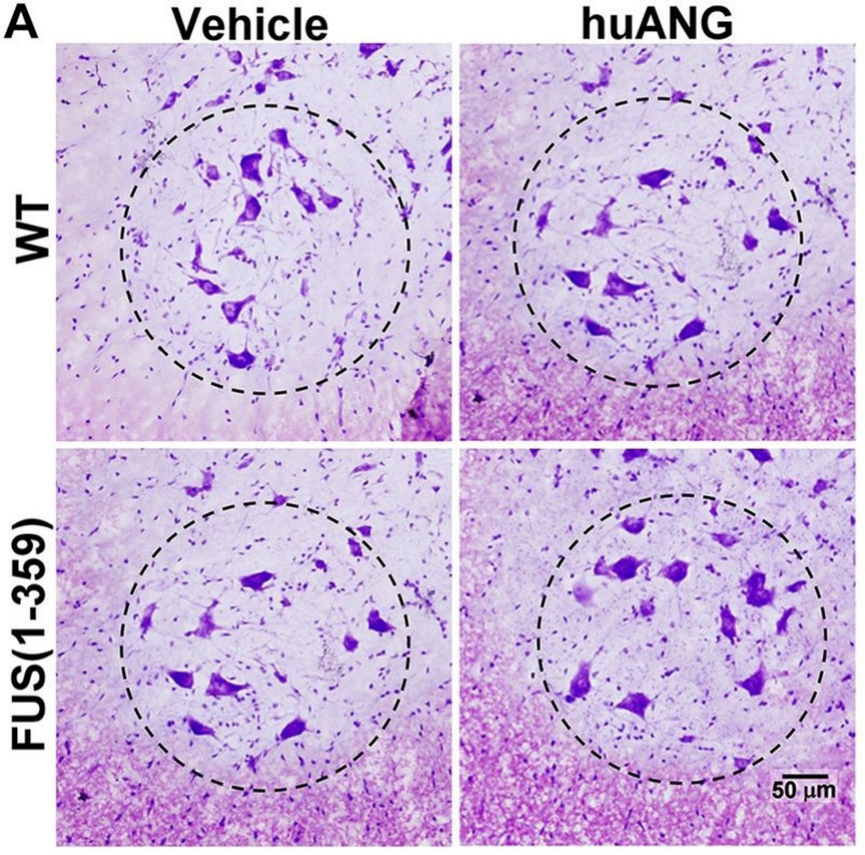
**huANG treatment did not affect expected motor neuron numbers.** (A) Representative images of Nissl-stained motor neurons in the ventral horn area of lumbar spinal cord tissue obtained at P70 from vehicle (left)- and huANG (right)-treated WT (top) and FUS (1-359) (bottom) mice. Dashed circle indicates ventral horn region used for quantification.

**Fig. 6A (corrected panel).**
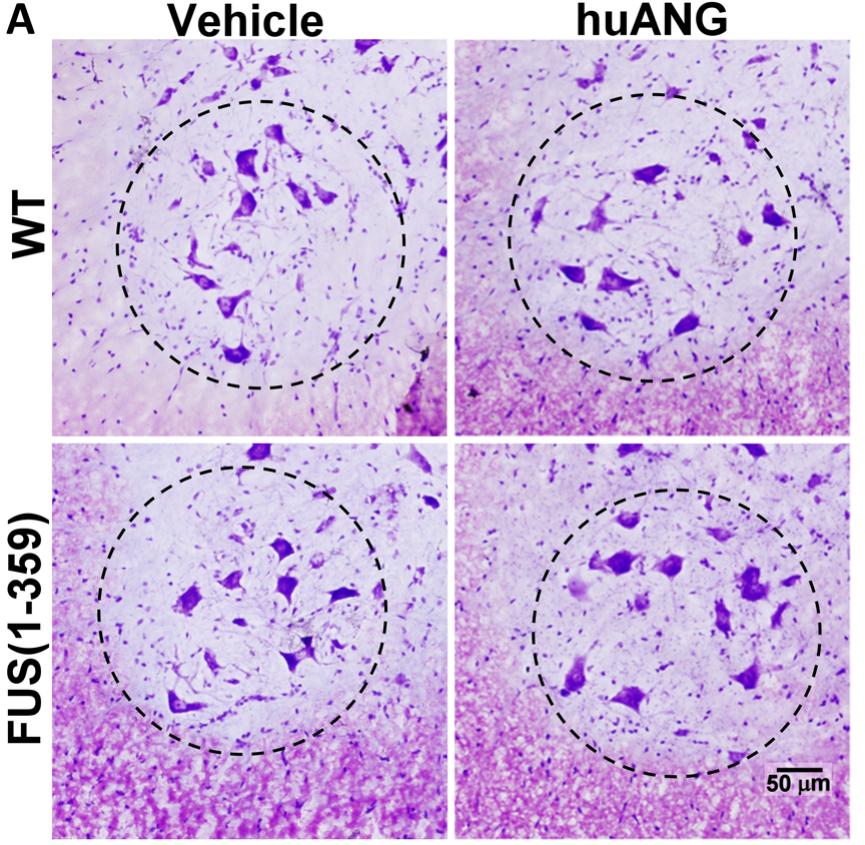
**huANG treatment did not affect expected motor neuron numbers.** (A) Representative images of Nissl-stained motor neurons in the ventral horn area of lumbar spinal cord tissue obtained at P70 from vehicle (left)- and huANG (right)-treated WT (top) and FUS (1-359) (bottom) mice. Dashed circle indicates ventral horn region used for quantification.

Both the online full-text and PDF versions have been updated and the authors apologise to readers for this error and any inconvenience it may have caused.

