# Supplementary material for: Vascular regression precedes motor neuron loss in the FUS (1-359) ALS mouse model
Source: Dis Model Mech. 2019 Aug 13;12(8):dmm040238. doi: 10.1242/dmm.040238 (PMC6737946; doi:10.1242/dmm.040238)
Supplement: Supplementary information [file dmm-12-040238-s1.pdf]

Table S1: Specific numbers of mice used for histological studies.

Tag #	Genotype	Sex	DOB	Age (days)	Nissl staining	Immunostaining PDCX-ASMA
0039	Tg	M	12/23/2015	50	✓	✓
0040	Tg	M	12/23/2015	50	✓	✓
0042	Tg	F	12/23/2015	50	✓	✓
0127	Tg	M	1/5/2016	50	✓	✓
0131	Tg	M	1/5/2016	50	✓	✓
0132	Tg	F	1/5/2016	50	✓	✓
0133	Tg	F	1/5/2016	50	✓	✓
0136	Tg	F	1/5/2016	50	✓	✓
0041	WT	M	12/23/2015	50	✓	✓
0043	WT	F	12/23/2015	50	✓	✓
0128	WT	M	1/5/2016	50	✓	✓
0134	WT	F	1/5/2016	50	✓	✓
0135	WT	F	1/5/2016	50	✓	✓
0138	WT	M	1/5/2016	50	✓	✓

Tag #	Genotype	Sex	DOB	Age (days)	Nissl staining	Immunostaining PDCX-ASMA
0004	Tg	M	11/25/2015	90	✓	X
0006	Tg	M	11/25/2015	90	✓	X
1525	Tg	F	9/6/2015	90	✓	✓
1978	Tg	F	11/24/2015	90	✓	✓
1979	Tg	F	11/24/2015	90	✓	✓
1981	Tg	F	11/24/2015	90	✓	✓
1982	Tg	F	11/24/2015	90	X	✓
1985	Tg	M	11/24/2015	90	X	✓
1989	Tg	F	11/24/2015	90	✓	X
1992	Tg	M	11/24/2015	90	✓	✓
1993	Tg	M	11/24/2015	90	✓	✓
1994	Tg	M	11/24/2015	90	✓	✓
0005	WT	M	11/25/2015	90	✓	✓
1524	WT	F	9/6/2015	90	✓	✓
1533	WT	M	9/6/2015	90	✓	✓
1980	WT	F	11/24/2015	90	✓	✓
1984	WT	M	11/24/2015	90	✓	✓
1986	WT	M	11/24/2015	90	✓	✓
1990	WT	F	11/24/2015	90	✓	✓
1995	WT	M	11/24/2015	90	✓	X

Disease Models & Mechanisms • Supplementary information

Tag #	Genotype	Sex	DOB	Age (days)	Nissl staining
0036	Tg	F	9/26/2014	120	✓
0047	Tg	M	10/5/2014	120	✓
0049	Tg	M	10/5/2014	120	✓
0050	Tg	M	10/5/2014	120	✓
0051	Tg	M	10/5/2014	120	✓
0052	Tg	M	10/5/2014	120	✓
0038	WT	F	9/26/2014	120	✓
0039	WT	F	9/26/2014	120	✓
0040	WT	F	9/26/2014	120	✓
0041	WT	F	9/26/2014	120	✓

Tag #	Genotype	Sex	DOB	Age (days)	Treatment	Nissl staining	Immunostaining PDCX-ASMA
0011	Tg	F	11/25/2015	70	1ug ANG	✓	✓
1926	Tg	F	11/7/2015	70	1ug ANG	✓	✓
1957	Tg	F	11/21/2015	70	1ug ANG	✓	✓
1962	Tg	F	11/21/2015	70	1ug ANG	✓	✓
1904	Tg	M	11/1/2015	70	1ug ANG	✓	✓
1905	Tg	M	11/1/2015	70	1ug ANG	✓	✓
1917	Tg	M	11/7/2015	70	1ug ANG	✓	✓
1922	Tg	M	11/7/2015	70	1ug ANG	✓	✓
0012	WT	F	11/25/2015	70	1ug ANG	✓	✓
1912	WT	F	11/7/2015	70	1ug ANG	✓	✓
1913	WT	F	11/7/2015	70	1ug ANG	✓	✓
1958	WT	F	11/21/2015	70	1ug ANG	✓	✓
1963	WT	F	11/21/2015	70	1ug ANG	✓	✓
1908	WT	M	11/1/2015	70	1ug ANG	✓	✓
1909	WT	M	11/1/2015	70	1ug ANG	✓	✓
1918	WT	M	11/7/2015	70	1ug ANG	✓	✓
1923	WT	M	11/7/2015	70	1ug ANG	✓	✓
1952	WT	M	11/21/2015	70	1ug ANG	✓	✓
0003	Tg	F	11/25/2015	70	Vehicle	✓	✓
1925	Tg	F	11/7/2015	70	Vehicle	✓	✓
1961	Tg	F	11/21/2015	70	Vehicle	✓	✓
1906	Tg	M	11/1/2015	70	Vehicle	✓	✓
1907	Tg	M	11/1/2015	70	Vehicle	✓	✓
1920	Tg	M	11/7/2015	70	Vehicle	✓	✓
1953	Tg	M	11/21/2015	70	Vehicle	✓	✓
1914	WT	F	11/7/2015	70	Vehicle	✓	✓
1915	WT	F	11/7/2015	70	Vehicle	✓	✓
1959	WT	F	11/21/2015	70	Vehicle	✓	✓
1964	WT	F	11/21/2015	70	Vehicle	✓	✓
1910	WT	M	11/1/2015	70	Vehicle	✓	✓
1911	WT	M	11/1/2015	70	Vehicle	✓	✓
1919	WT	M	11/7/2015	70	Vehicle	✓	✓
1924	WT	M	11/7/2015	70	Vehicle	✓	✓

Disease Models & Mechanisms • Supplementary information

Table S2: Specific numbers of mice used for lifespan studies.

Tag #	Genotype	Sex	Treatment	DOB
434	Tg	F	huANG	1/19/2015
435	Tg	F	huANG	1/19/2015
436	Tg	F	huANG	1/19/2015
437	Tg	F	huANG	1/19/2015
451	Tg	M	huANG	1/24/2015
452	Tg	M	huANG	1/24/2015
453	Tg	M	huANG	1/24/2015
614	Tg	F	huANG	2/28/2015
615	Tg	F	huANG	2/28/2015
616	Tg	F	huANG	2/28/2015
765	Tg	F	huANG	3/15/2015
769	Tg	F	huANG	3/15/2015
747	Tg	M	huANG	3/16/2015
749	Tg	M	huANG	3/16/2015
750	Tg	M	huANG	3/16/2015
1128	Tg	F	huANG	5/26/2015
1129	Tg	F	huANG	5/26/2015
1134	Tg	M	huANG	6/2/2015
1213	Tg	F	huANG	6/18/2015
1215	Tg	M	huANG	6/18/2015
1348	Tg	M	huANG	7/1/2015
1379	Tg	M	huANG	7/19/2015
447	Tg	M	PBS	1/24/2015
786	Tg	M	PBS	3/15/2015
788	Tg	F	PBS	3/17/2015
790	Tg	F	PBS	3/17/2015
792	Tg	F	PBS	3/23/2015
793	Tg	F	PBS	3/23/2015
1019	Tg	M	PBS	5/7/2015
1020	Tg	M	PBS	5/7/2015
1027	Tg	F	PBS	5/7/2015
1028	Tg	F	PBS	5/7/2015
1061	Tg	M	PBS	5/12/2015
1054	Tg	F	PBS	5/19/2015
1055	Tg	F	PBS	5/19/2015
1132	Tg	F	PBS	5/26/2015
1137	Tg	F	PBS	6/2/2015
1138	Tg	F	PBS	6/2/2015
1139	Tg	F	PBS	6/2/2015
1140	Tg	F	PBS	6/2/2015
1142	Tg	F	PBS	6/2/2015
1214	Tg	F	PBS	6/18/2015
1205	Tg	M	PBS	6/19/2015
1209	Tg	M	PBS	6/18/2015
1339	Tg	M	PBS	7/1/2015
1362	Tg	M	PBS	7/16/2015
1362	Tg	M	PBS	7/16/2015

Disease Models & Mechanisms • Supplementary information
